# Trying on tRNA for Size: RNase P and the T-box Riboswitch as Molecular Rulers

**DOI:** 10.3390/biom6020018

**Published:** 2016-04-01

**Authors:** Jinwei Zhang, Adrian R. Ferré-DAmaré

**Affiliations:** 1 Laboratory of Molecular Biology, National Institute of Diabetes and Digestive and Kidney Diseases, 50 South Drive, Bethesda, MD 20892, USA; 2 Laboratory of RNA Biophysics and Cellular Physiology, National Heart, Lung and Blood Institute, 50 South Drive, Bethesda, MD 20892, USA

**Keywords:** RNA structure, molecular rulers, tRNA, RNase P, T-box, riboswitch, tRNA elbow, interdigitated T-loops, noncoding RNA, noncanonical base pairs

## Abstract

Length determination is a fundamental problem in biology and chemistry. Numerous proteins measure distances on linear biopolymers to exert effects with remarkable spatial precision. Recently, ruler-like devices made of noncoding RNAs have been structurally and biochemically characterized. Two prominent examples are the RNase P ribozyme and the T-box riboswitch. Both act as molecular calipers. The two RNAs clamp onto the elbow of tRNA (or pre-tRNA) and make distance measurements orthogonal to each other. Here, we compare and contrast the molecular ruler characteristics of these RNAs. RNase P appears pre-configured to measure a fixed distance on pre-tRNA to ensure the fidelity of its maturation. RNase P is a multiple-turnover ribozyme, and its rigid structure efficiently selects pre-tRNAs, cleaves, and releases them. In contrast, the T-box is flexible and segmented, an architecture that adapts to the intrinsically flexible tRNA. The tripartite T-box inspects the overall shape, anticodon sequence, and aminoacylation status of an incoming tRNA while it folds co-transcriptionally, leading to a singular, conditional genetic switching event. The elucidation of the structures and mechanisms of action of these two RNA molecular rulers may augur the discovery of new RNA measuring devices in noncoding and viral transcriptomes, and inform the design of artificial RNA rulers.

## 1. Trying on tRNA for Size

Transfer RNAs (tRNAs) were the first discovered noncoding RNAs, and also the first to demonstrate that RNA can adopt elaborate three-dimensional structures [1,2]. Both the overall fold and local features of tRNA (such as the anticodon, elbow, and termini) are required for it to carry out its key adaptor function on the ribosome [3]. In doing so, tRNAs act as physical and informational conduits that connect the RNA world to that of proteins.

**Figure 1:**
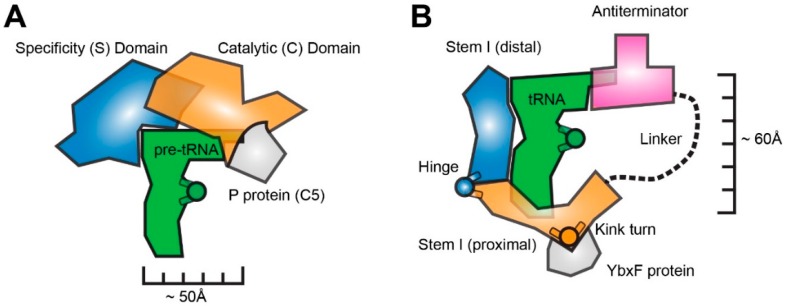
Cartoon representations of the overall domain organization of bacterial RNase P ribozymes (**A**) and T-box riboswitches (**B**). Circles with two projecting arms denote location of molecular “hinges”. Associated proteins that assist the RNA functions are also indicated.

The canonical L-shaped architecture of tRNA results from the association of four structural elements with conserved dimensions [1,2,4]. The aminoacyl acceptor stem is comprised of seven base pairs, and bears four unpaired nucleotides (-NCCA) at its 3' terminus. The anticodon stem-loop (ASL) consists of five base pairs closed by a heptanucleotide loop. The D stem-loop (DSL) consists of four base pairs and a 8–9 nucleotide loop. The T stem-loop (TSL) has five base pairs and a heptanucleotide loop. Therefore, and despite the sequence variation among tRNAs, the differing patterns of post-transcriptional modifications, and the differences in the variable (V) loop, most tRNAs are near-isosters. The common molecular dimensions of tRNA are obviously essential for efficient and kinetically equivalent passage of different aminoacyl- (and peptidyl-) tRNAs through the translating ribosome [3]. In addition, the structural conservation of tRNAs enables other molecules to identify these noncoding RNAs independently from their primary sequence. In particular, the conservation of the lengths of its two near-orthogonal helical stacks (the “upper” or TSL-Acceptor Stem stack and the “lower” or ASL-DSL stack) enables tRNA recognition by several RNAs, ribonucleoproteins, and proteins that function as “molecular rulers” (Figure 1). Any molecule that measures distances on another molecule can be considered a molecular ruler. In this review, we limit the definition to molecular devices made of biological polymers that measure linear distances on a target macromolecule in *trans*. We note that some molecular rulers are more accurately characterized as “molecular calipers” as they use two flanking domains to clamp onto either end of their target molecules.

## 2. RNase P is a Rigid, Pre-Folded tRNA Ruler

**Figure 2:**
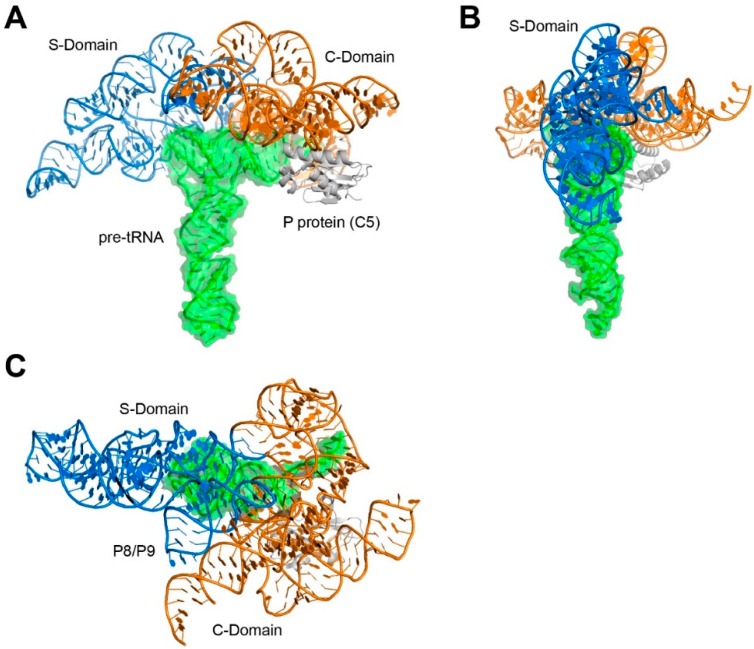
Structure of a bacterial RNase P holoenzyme in complex with its pre-tRNA substrate. (**A**) Front view; (**B**) side view; and (**C**) top view. PDB ID: 3Q1Q [15]. All molecular graphics were generated with PyMol [18].

The ribonuclease (RNase) P ribozyme was the first functionally and structurally characterized example of an RNA molecular ruler (and caliper) that measures another RNA [5,6,7,8]. This ribozyme binds to the TSL-Acceptor Stem stack of all pre-tRNAs, and measures a precise, invariant distance from the tRNA elbow to cleave its substrates and yield mature tRNA 5' ends [9,10,11,12,13,14]. Reliance on distance measurement, rather than sequence-specific interactions, allows one ribonuclease to process all pre-tRNAs, despite their differences in sequence and post-transcriptional modifications. RNase P consists of two similarly-sized domains: the specificity domain (S domain) and the catalytic domain (C domain). Extensive covalent and non-covalent interactions couple the two domains to yield a dome-like structure that overhangs the pre-tRNA substrate (Figure 1 and Figure 2) [15,16]. The two domains are part of one continuous RNA chain, and a coaxial stack extends between C domain helix P6 and S domain helix P7. Numerous tertiary interactions interweave the two domains. Only minimal differences exist between the crystal structures of the free RNA and the pre-tRNA-bound holoenzyme, indicating that this is a rigid, preformed molecular ruler [15,17]. RNase P structure, substrate recognition, and catalysis are discussed elsewhere in this Special Issue.

## 3. The T-box Riboswitch is a Flexible, Segmented tRNA Ruler

**Figure 3:**
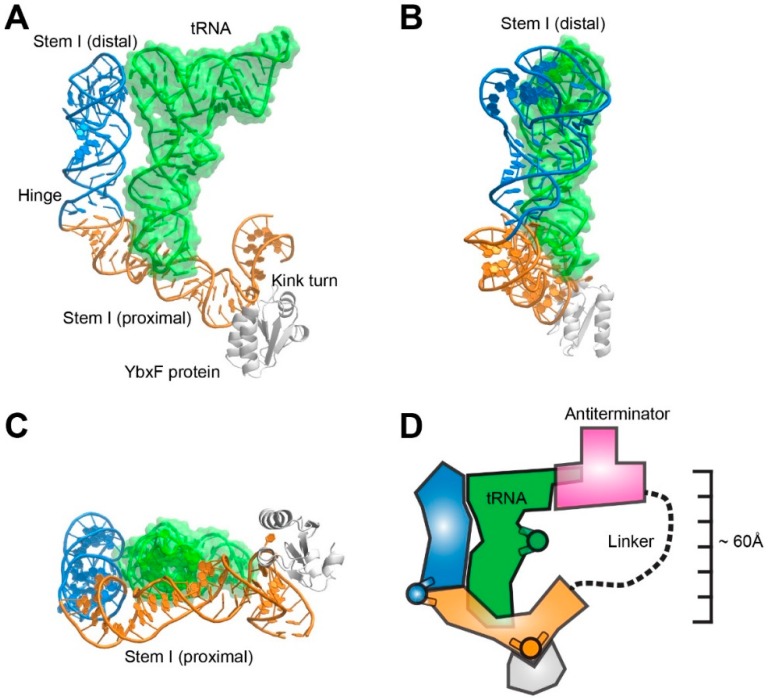
Structure of a T-box riboswitch Stem I domain in complex with its cognate tRNA. (**A**) Front view; (**B**) side view; (**C**) top view; and (**D**) cartoon schematic of a proposed structural model of a full-length T-box-tRNA complex. PDB ID: 4LCK [26].

T-box riboswitches are bacterial gene-regulatory mRNA domains that control transcription in response to the intracellular abundance of specific amino acids [19,20,21]. They do so by detecting the aminoacylation status of tRNAs as a proxy. These riboswitches have a phylogenetically-conserved structure comprised of two structurally- and functionally-separable domains, the 5' Stem I and the 3' Antiterminator, which are connected by a linker of variable length and sequence (Figure 1B and Figure 3) [22,23]. The Stem I domain is necessary and sufficient for specific recognition of one tRNA (some T-boxes may bind more than one tRNA [24,25]), and carries a trinucleotide sequence that is complementary to the anticodon of its cognate tRNA (thus, this “specifier” trinucleotide is, in effect, the codon trinucleotide) [25,26]. Once Stem I binds to a tRNA, the Antiterminator domain evaluates its aminoacylation status, and terminates transcription if the tRNA is charged [20,27].

### 3.1. T-box Stem I is A Flexible, Hinged Ruler That Molds to tRNA.

The crystal structures of cognate complexes between T-box Stem I domains and tRNA revealed that this riboswitch structural element has distinct proximal and distal subdomains [26,28]. The subdomains directly recognize the anticodon and the elbow, respectively, of the cognate tRNA (Figure 3). In so doing, the Stem I domain measures a distance of ~60 Å along the ASL-DSL coaxial stack. The molecular ruler character of the T-box Stem I is supported by the observation that insertions with integral numbers of helical turns into Stem I can compensate for corresponding insertions in the tRNA ASL [28].

Unlike the relatively rigid TSL-Acceptor Stem stack measured by RNase P, the ASL-DSL stack of tRNA contains a hinge at the noncanonical 26•44 base pair that forms the junction between the D and anticodon stems [29,30]. tRNAs can flex about this hinge by as much as 70° during transit through the ribosome, helping retain contacts with both ribosomal subunits as these move relative to each other during translation [31]. The T-box has evolved to accommodate this inherent, functionally-essential flexibility of tRNA, and possibly also structural differences among different tRNA subtypes [32]. Crystallographic analysis shows that the *Oceanobacillus iheyensis glyQ* T-box Stem I captures its cognate tRNA^Gly^ in a slightly more bent conformation than that of a ribosome-bond P/P state tRNA [26]. It is bent by 20° relative to the structure of free tRNA^Phe^, while a P/P state tRNA is only bent by 12°.

Comparisons of structures of an isolated Stem I distal fragment and of complexes between full Stem I domains and tRNA reveal bending about a hinge in the riboswitch that avoids steric clash with the incoming tRNA [26,33]. Interestingly, the sequence and structure of the Stem I hinge exhibit species-specific differences. While the *Geobacillus kaustophilus glyQ* T-box employs a C-loop motif (or CUC bulge) with an extruded nucleotide (C74), the *O. iheyensis glyQ* T-box features a simpler hinge characterized by a two-nucleotide insertion (A29-U30) on the 5' RNA strand [19,26,33]. Disruption of the Stem I hinge severely compromises tRNA binding, as evidenced by a 12-fold reduction in binding affinity measured by isothermal calorimetry from a Δ29-30 deletion in *O. iheyensis* T-box [26]. Similarly, replacing the C-loop in *G. kaustophilus* T-box with a Watson-Crick pair renders tRNA binding undetectable in a gel-shift assay [28].

At the proximal end of most T-box Stem I domains is a conserved Kink turn (K-turn) [34,35,36]. This is a widespread RNA structural motif that bends the helical trajectory of the RNA by 120° and is the binding site for proteins such as YbxF (of the L7Ae family) [37]. It remains unclear why the K-turn is required for T-box function *in vivo* [35], as it does not appear to contribute to Stem I-tRNA binding [26,33]. The crystal structure of the *O. iheyensis* Stem I-tRNA complex suggests that, although the K-turn is located in the Stem I domain, its role may be to facilitate coupling of initial tRNA recognition by this domain and subsequent evaluation of tRNA aminoacylation status by the Antiterminator domain [26]. The K-turn, and the proteins that stabilize its characteristic sharp kink may be responsible for redirecting the path of the polynucleotide chain and projecting the 3' Antiterminator domain of the T-box to the vicinity of the tRNA 3' end. The tripartite recognition of the tRNA elbow, anticodon, and 3' end by a single T-box RNA calls for a U-shaped overall architecture [19,26,27]. Since coaxial helical stacks dominate RNA structures, multiple bends are required to construct a shape to track the tRNA. The mild bend of the Stem I hinge and the sharp kink of the K-turn together contribute to this in the case of T-box riboswitches.

### 3.2. The T-box Inter-Domain Linker and Antiterminator: A Molecular Lasso?

The antiterminator domain is responsible for making the gene-regulatory decision of the T-box, and is composed of two helices and a bulge [21,22,23]. Biochemical and biophysical analyses suggest that it sterically senses the aminoacylation status of tRNA, and couples this readout with the formation of mutually exclusive terminator or antiterminator RNA structures [27]. The dichotomy of two competing RNA structures with opposite gene expression outcomes, formation of which is a function of tRNA aminoacylation state is, thus, the basis of the T-box genetic switch.

Stem I substitutions that disrupt tRNA binding disable T-box function *in vivo* and *in vitro* [26,27,35]. This suggests that the antiterminator domain cannot bind the tRNA 3'-end unassisted. Instead, its engagement with tRNA has to be facilitated by local concentration effects and, possibly, enhanced geometrically by the directional bend of the Stem I K-turn.

The intervening sequences between the Stem I and antiterminator domains are variable and appear to be mostly single-stranded and unstructured [21,22,23]. If the inter-domain linker is indeed unstructured, its role resembles that of a lasso (“thrown” by Stem I) that is projected towards its target, the tRNA 3'-end. Then the primary contributions of Stem I are initial tRNA recognition and molecular tethering. The idea of an unstructured linker is largely consistent with the finding that significant helical extensions to the tRNA Acceptor Stem are tolerated by the T-box, albeit in a helical phase-sensitive manner [38]. However, the inter-domain linker invariably contains a Stem III structure, and in the vast majority of the T-boxes, also contains conserved Stem II and Stem IIA/B pseudoknot structures instead of the presumably single-stranded linker found in glycine-responsive T-boxes [22,23]. The structure and function of these intervening elements remain unknown, but there are several possibilities. First, they could conceivably act as a structured molecular ruler that accurately positions and orients the antiterminator domain to interact with tRNA 3' end. Second, these structures could make additional contacts to the tRNA, such as the variable stem loop found in some tRNAs. These additional contacts may compensate for the lack of tRNA elbow-contacting Stem I distal region in some T-boxes (in particular those that regulate translation initiation rather than transcription termination), or provide additional tRNA specificity [39]. Third, these intervening structures or sequences may pause the transcribing RNA polymerase to extend the time window available for tRNA to fully engage with Stem I. This is plausible because most T-boxes operate co-transcriptionally as tRNA progressively engages them *via* multiple points of contact [40,41,42].

**Figure 4:**
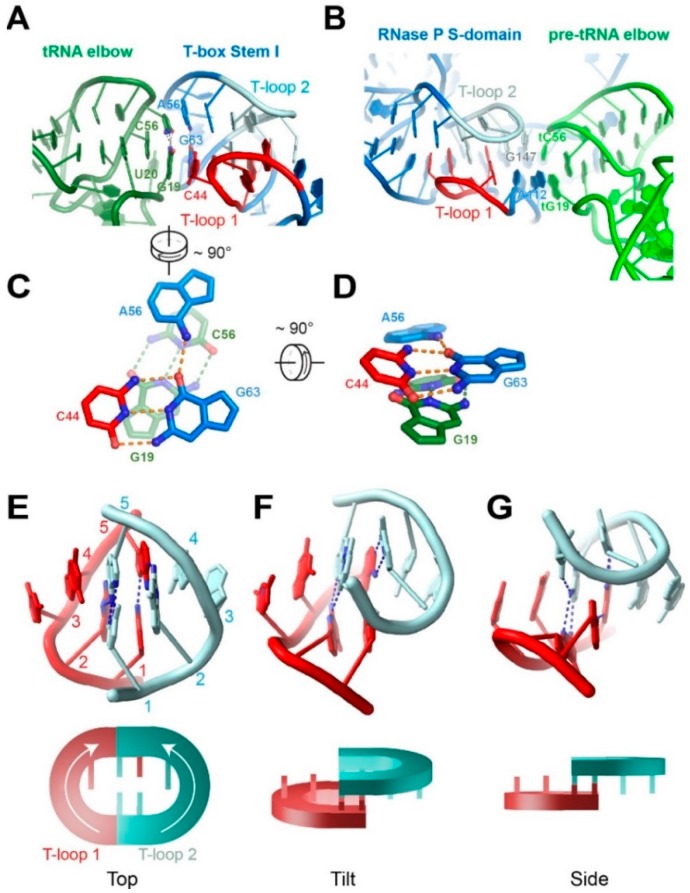
The interdigitated T-loops mediate the recognition of tRNA elbow structure. (**A**) tRNA elbow recognition by the T-box stem I domain (PDB ID: 4LCK) [26]; (**B**) pre-tRNA elbow recognition by the RNase P S domain (PDB: 3Q1Q) [15]; (**C**,**D**) two orthogonal views from (A), of the stacking interface between the T-box base triple and the tRNA tertiary base pair. Hydrogen bonds are indicated by dashed lines; and (**E**–**G**) three views of the structure of the interdigitated T-loop motif. Nucleotides of each T-loop are numbered 1–5 in the direction of 5' to 3'. Cartoon representations of the motif are shown beneath the structural models.

T-boxes bind specifically to their cognate tRNAs, and structural analyses revealed that they measure tRNAs from the anticodon to the elbow, along the ASL-DSL coaxial stack orthogonal to the tRNA edge (TSL-Acceptor Stem coaxial stack) measured by RNase P. Many interesting parallels and distinctions exist between how these two noncoding RNAs size up tRNA. Functionally speaking, the RNase P endonucleolytically processes all pre-tRNA substrates irrespective of their amino acid specificity, contributing to the formation of mature tRNAs. The T-box RNA recognizes one or a few tRNAs (as they directly inspect the anticodon), evaluates their aminoacylation status to sense amino acid starvation, and governs the transcription or translation of downstream genes involved in amino acid metabolism. Structurally speaking, both the RNase P and the T-box recognize the overall structure of tRNA and share a structural motif that they use to recognize the tRNA (or pre-tRNA) elbow (Figure 1 and Figure 4).

## 4. How to Make a Molecular Ruler with RNA

A canonical molecular ruler, like the actual tool, is composed of a relatively rigid body of a pre-defined length and shape, anchor points to establish register with the target molecule and, frequently, an effector domain located at one end of the ruler. Directed or aided by the molecular ruler, the effector performs a local transaction with the target molecule, which can take the form of recognition, association, or chemical catalysis with downstream biological significance.

### 4.1. Shape, Phase, and Register

RNAs make for structurally and functionally robust molecular rulers. The A-form RNA duplex is not only thermodynamically more stable, but also mechanically more stiff (or less compliant) than DNA duplexes. Under physiological conditions, RNA duplexes exhibit persistence lengths of 600–800 Å compared to 450–500 Å for DNA duplexes [43,44]. The strong enthalpic drive to anneal complementary strands coupled with axial stiffness of RNA duplexes makes them excellent scaffolds to organize large ribonucleoprotein complexes, and also accurate, predictable molecular rulers. Compared to proteins, the coupling of stacking interactions and electrostatic repulsion of neighboring phosphates produces a very stiff molecule [45]. Indeed, most RNA structures solved to date (except for the ribosomal RNAs) are oblate, rather than quasi-spherical as most proteins. The elongated shape of most RNAs is inherently suitable for measuring distances.

In addition to forming stable structures, RNA duplexes project functional groups in a predictable and phase-sensitive manner. As a full helical turn in A-form RNA duplex consists of 11 base pairs, each rung of the ladder, or base pair, is rotated ~32.7° relative to the next, making available a diverse set of approaching angles. Remarkably, a number of RNA structural motifs, such as the widespread C-loop motif (or loop C motif) and loop E motif (or bulged G motif), substantially alter the helical twist of RNA duplexes in predictable manners [46,47]. These motifs adeptly solve the phase problem that can arise due to the discontinuous helical rise, allowing multivalent interactions to engage simultaneously. Indeed, both C-loop and loop E motifs occur frequently in T-box Stem I, presumably to adjust the helical twist and phase to allow its bipartite binding to both the tRNA anticodon and the elbow.

Helical extensions in the tRNA ASL or Acceptor Stem are accommodated by a similarly extended T-box Stem I or a flexible Stem I-linked antiterminator, respectively, both in a phase-sensitive manner [28,38]. Insertion of five base pairs nearly inverts the phase, resulting in loss of interaction whereas insertion of 11 base pairs restores binding. The projection of a functional group from RNA duplexes could originate intrahelically from either the major groove (Hoogsteeen edge) or minor groove (sugar edge), or extrahelically from a flipped nucleotide. Pyrimidines, in particular, uridines, are common flipped nucleotides to engage tertiary interactions with other RNAs or proteins, such as RNA modification enzymes [48,49]. Post-transcriptional modifications to the RNA alter the energetics and thus tendencies of extrahelical flipping—with, *e.g*., dihydrouridines favoring flipping and pseudouridines disfavoring it [50].

In addition to appropriately shaped ruler bodies with versatile positions and angles to project functional groups, effective molecular rulers must also establish register relative to prominent landmarks on the molecules being measured. In this respect, both the RNase P and the T-box Stem I use a remarkably similar strategy to establish points of contact to the tRNA (or pre-tRNA). At one end of the ruler, 3–4 primarily Watson-Crick base pairs set up a sequence-specific anchor between single-stranded regions (presented from overhangs, loops, bulges, *etc*.) from each RNA. tRNA 3' end pairs with complementary nucleotides in the L15 loop or P15 hairpin of the RNase P [15]. On the other end of the ruler, the characteristically flat tRNA elbow structure is recognized by a recurring RNA motif formed by the interdigitation of two pentanucleotide T-loops [51]. This interaction involves stacking contacts that are not sequence-specific but are, nonetheless, sequence-biased in favor of bicyclic purine nucleobases for enhanced stacking [26,52].

### 4.2. The Interdigitated T-Loop Motif

The interdigitated T-loop motif has recently been recognized as a structural motif that specifically recognizes the tRNA elbow in three separate, evolutionarily-unrelated instances—the RNase P, the T-boxes, and the L1 stalk of the ribosome [51,52,53]. It is formed by the head-to-tail interdigitation of two pentanucleotide T-loops that pair and interlock, filling the stacking gap present between the fourth and fifth nucleotides of each T-loop motif (Figure 4) [26,33,52]. The resulting structure features six extensively base-paired and densely-stacked layers of nucleotides that emanate from two central base triples. Remarkably, none of the base-pairing interactions found in RNase P’s interdigitated T-loops are Watson-Crick pairs. The homologous structure employed by the T-box Stem I has but one Watson-Crick pair that forms part of the base triple that the RNase P lacks (Figure 4C). The paucity of canonical base pairs that stitch together this structural motif shows that RNA nucleotides can effectively employ Hoogsteen and sugar-edge contacts to build sturdy structural motifs that essentially circumvent the use of Watson-Crick edges used in A-form duplexes. This fact also makes it more challenging to predict these interactions *in silico* as canonical Watson-Crick sequence complementarity (including wobble pairs) drives most secondary structure prediction algorithms.

Due to the prevalence of tRNA and tRNA-like structures in the cell, it is conceivable that additional cellular noncoding RNAs and viral RNAs may have evolved devices that manipulate these structures, in ways akin to the RNase P and T-box systems. The consensus choice of the interdigitated T-loops motif to recognize the tRNA elbow makes it an attractive target to search for in the noncoding and viral transcriptomes for novel RNAs that potentially bind tRNA or tRNA-like structures. Interestingly, a number of nematode and metazoan mitochondrian tRNAs lack either the DSL, the TSL, or both, making them elbow-less [54,55,56]. In these organelles, the RNase P and ribosomal L1 stalk correspondingly lack the sequences necessary for formation of the interdigitated T-loops [52]. There are at present no known T-boxes that operate in the mitochondria. These observations suggest the co-evolution of tRNA structure with their RNA molecular rulers.

### 4.3. Non-tRNA Substrates of RNA Molecular Rulers

The molecular ruler nature of these RNA devices imply that other RNA molecules that satisfy the measurements of the ruler will also gain access to the ruler-associated functions. The ruler in the RNase P is designed to fit the TSL-Acceptor Stem of pre-tRNA. Thus, any RNA structures that present such a structure will be recognized and cleaved. Prominent non-tRNA substrates of RNase P include the transfer-messenger RNA (tmRNA) [57], 4.5S RNA of the signal-recognition particle [58], viral RNAs such as hepatitis C virus (HCV) [59], long noncoding RNAs, such as MALAT-1 and Menβ, *etc.* [60]. As expected, most of these RNA substrates present tRNA-like structures. Viral RNAs, prominently those with internal ribosome entry sites (IRESs), go to great lengths to create tRNA-like structures (using for instance pseudoknots) to hijack cellular machineries including tRNA rulers, for viral replication and translation, *etc*. [61,62].

Interestingly, not all of these RNase P substrates, such as 4.5S RNA, have immediately recognizable tRNA-like structures. In light of the recent proposal that tRNAs, ribosomal RNAs, viral RNAs, *etc*., may have descended from the same group of ancient stem-loop hairpin RNAs, RNase P may detect certain common RNA structural features that have not been well recognized [63,64]. Detailed structural studies will be necessary to understand the 3D structure of these substrates and how they satisfy the stipulated measurements by RNase P. Of particular interest will be whether these alternative RNA species present a flat structure that resembles the tRNA elbow. It appears that the tRNA D-loop is dispensable but the T-loop or a similar loop structure is needed to trigger RNase P cleavage. The principles of pre-tRNA recognition by RNase P have been widely harnessed to selectively degrade selected cellular mRNAs that pair with an exogeneous guide RNA to form a pre-tRNA-like structure. Details of RNase P-mediated knockdown of target RNAs are summarized in an accompanying article in this Special Issue [65].

It is presently unclear if the T-box riboswitches interact with natural, non-tRNA substrates. The fact that T-boxes recognize essentially all the defining features of tRNA—the anticodon, elbow, and 3'-end—places a more stringent set of requirements on the structure and features that a non-tRNA must mimic.

## 5. RNase P and T-box RNAs *vs.* Other Molecular Rulers

### 5.1. Diverse Definitions and Types of Molecular Rulers

The term “molecular ruler” has been used to convey several distinct meanings in various biological contexts. A number of intracellular, extracellular, and membrane-embedded or -anchored supramolecular structures are assembled from smaller building blocks [66]. In one mechanism, exemplified by microtubules assembled from tubulin, lengths are kinetically determined by simultaneous, opposing action of polymerization and depolymerization. In contrast to these dynamically balancing rulers, other systems employ specialized ruler molecules that gauge the overall length or number of repeat units during polymerization or self-assembly and prevent further chain growth to give rise to desired lengths. The evolutionarily-related, functionally-divergent bacterial flagella and injectisomes use secreted ruler proteins to determine needle length [67,68]. The tail lengths of lambda and T4 bacteriophages are determined by an extended ruler protein that is tethered adjacent to the initiator complex and is as long as the tail [69,70]. Other known mechanisms of length determination exploit limiting supplies of precursor for assembly (e.g., centrosome biogenesis), or gradients of diffusing molecules (e.g., mitotic spindle assembly) [66].

The first example of a molecular ruler made of RNA came from pioneering work on the Tobacco Mosaic Virus (TMV). RNA structures play key roles in the assembly and length determination of the rod-shaped viral particle that envelops a single-stranded RNA corkscrew. A traveling RNA stem loop guides the assembly of two-layered coat protein disks (34 units) while the overall length of the genomic RNA (~6400 nucleotides) dictates the total number of coat proteins (2130 units) and, thus, the size of the viral particle—300 nm in length and 18 nm in diameter [71]. In this case, length is measured not linearly, but on a helical trajectory along which each coat protein contacts a RNA trinucleotide.

### 5.2. RNase P and T-box are Trans-Acting Molecular Calipers

In most of the preceding examples, the “ruler” molecule serves more as a template or guide instead of a measuring device, and often the length of the ruler molecules are the same as the structures whose lengths are to be determined. These rulers function in the biogenesis or maintenance of structures of prescribed dimensions. The cases of RNase P and T-box riboswitches are different from those systems in several respects. First, instead of measuring along the entire length of the subject (e.g., the TMV case), the RNase P and T-box Stem I engage the tRNA (or pre-tRNA) arms primarily at their ends. This makes them more like molecular “calipers” than rulers. Compared to rulers, end-clamping calipers are more easily reconfigured to provide broader compatibility, as they do not recognize the middle sections of the target. For the same reason, calipers may exhibit increased chances for cross-reaction with unintended molecular targets. Second, instead of initially guiding the formation of desired lengths (*cis*-acting in a way), RNase P and T-box are *trans*-acting devices that gauge the dimensions of objects that they encounter and the molecular calipers serve to selectively recognize and capture the anticipated targets. Third, both RNase P and T-boxes are considerably larger than the tRNA (or pre-tRNA) they measure. In addition to the tRNA-recognition domains, RNase P and the T-boxes possess functional domains that carry out ribozyme catalysis and conditional genetic switching, respectively.

The structural rigidity of the RNase P and flexibility of the T-boxes likely reflect their distinct cellular functions. As a multiple-turnover ribozyme tasked with processing tens of thousands of pre-tRNAs, RNase P needs to be efficient and accurate. Due to the relative rigidity of the tRNA TSL-Acceptor Stem stack, the pre-organized structure of RNase P is poised to quickly clamp both ends of this arm. The relatively inflexible design ensures selection of the correct cleavage site, and the minimal contacts to the mid-section of the tRNA arm may facilitate rapid product release after cleavage. The T-box, on the other hand, is a single-turnover genetic switch whose imperative is to balance sensitivity, accuracy, and range of response. The intrinsic flexibility and variability of the tRNA ASL-DSL stack necessitates a more adaptable molecular caliper. The segmented, hinged design can better leverage induced fit to achieve increased affinity and avidity of binding, thus boosting its sensitivity to detect low levels of amino acid starvation. The single-turnover nature of the T-box makes less consequential the extent or rate of tRNA release from the T-box, once the genetic decision is rendered.

### 5.3. Protein Molecular Rulers that Measure RNA

**Figure 5:**
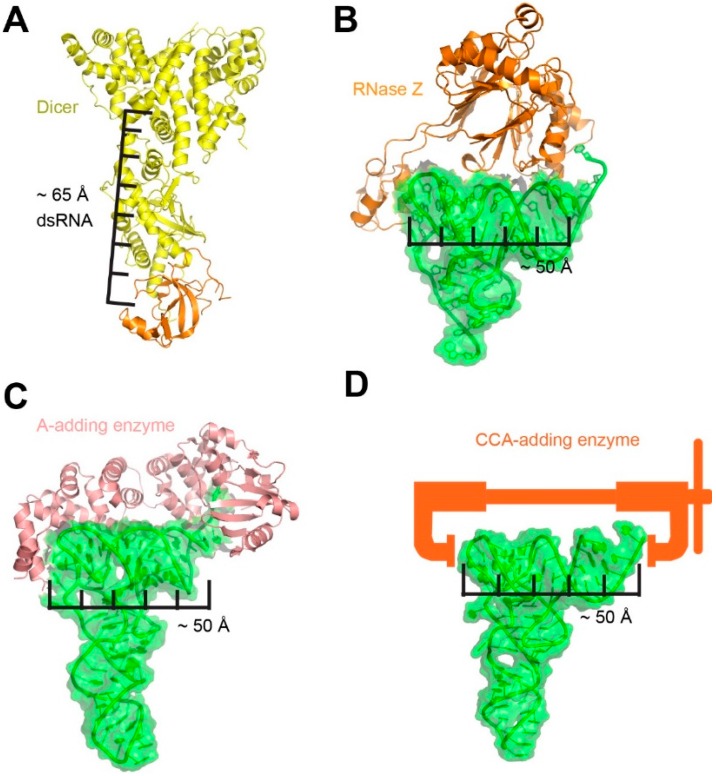
Selected examples of proteins measuring distances on RNA structures. (**A**) Dicer measurement of double-stranded RNAs (dsRNAs). PDB ID: 2FFL [72]; (**B**) RNase Z measurement of pre-tRNAs. PDB ID: 4GCW [74]; (**C**) A-adding enzyme measurement of 3'-CC-containinng pre-tRNAs. PDB ID: 4X0B [75]; and (**D**) CCA-adding enzyme acting as a molecular vise that mechanically inspects tRNA structural integrity [76].

In contrast to the paucity of known examples of molecular rulers or calipers made of RNA, numerous RNA-binding proteins use a combination of localized feature recognition and geometric measurement to carry out specific recognition or transformation of the RNA substrate. In RNA interference, specialized endonuclease Dicer measures 65 Å from its RNA binding PAZ domain to its RNase III catalytic site, a distance that spans 20–25 base pairs along the RNA duplex, thus dictating the lengths of its siRNA and miRNA products (Figure 5A) [72]. RNase Z, the tRNA maturase for CCA-less pre-tRNAs, clamps the tRNA elbow and recognizes the pre-tRNA 3' region to remove extraneous 3' tails. It appears to use both distance measurement and local feature recognition (in particular, 3' CCA as a primary antideterminant) to determine proper cleavage site (Figure 5B) [73,74]. After RNase Z trimming, 3' CCA is added either by a single CCA-adding enzyme, or by the sequential action of a CC-adding enzyme and an A-adding enzyme. In the latter system, the A-adding enzyme establishes a single, specific register at the tRNA elbow using its C-terminal tail domain. The nucleotidyl transferase domain is placed a fixed distance away such that only a CC-containing tRNA 3' end can reach it and be adenylated (Figure 5C) [75]. In an intriguing example, the CCA-adding enzyme not only measures the distance from the tRNA elbow to its 3' end, but further gauges the compressive strength of the TSL-Acceptor Stem stack as a quality control device [76,77]. While structurally sound tRNAs spring out of the enzyme's grasp when compressed, defective tRNAs are collapsed by the molecular vise and are retained on the enzyme (Figure 5D). The 3' strand of the Acceptor Stem is mechanically sheared to form a 3-nt bulge and reforms a duplex with the 5' strand in a shifted register, thus allowing enzymatic addition of a second CCA tag as a mark for degradation [76].

### 5.4. Evolution and Application of RNA Molecular Rulers

A number of advantages and utilities drive the evolution and fixation of RNA molecular rulers and calipers, which by definition rely more on geometric measurement rather than highly stereospecific local recognition. First, molecular rulers and calipers can be broadly compatible with a large group of self-similar molecular entities, such as tRNAs that carry different anticodons and amino acids but, nonetheless, share the same set of overall geometric characteristics. Second, molecular rulers and calipers are strongly preferred when the desired site of transaction (such as cleavage or chemical modification) lacks sufficient local structural specificity for *in situ* recognition. Instead, precise delivery of an effect or transformation is accomplished by the use of a secondary site where specific recognition is more easily achieved, from which a defined distance is measured to locate the primary site. Thirdly, molecular rulers and calipers tend to be adaptable and configurable. By altering certain structural parameters, many molecular rulers and calipers can easily adapt to work with newly-evolved, or artificially-introduced partners. The structural and mechanistic understanding of these devices can, then, inform design and engineering of molecular devices that elicit desired biological outcomes. For instance, the RNase P is already widely applied to target selected cellular mRNAs for cleavage and degradation [78]. The T-box riboswitches could be engineered as “anti-tRNAs” that specifically recognize cellular tRNAs in organisms that lack them. Conceivably, the T-boxes could be conjugated to other functional RNA domains such as ribozymes or RNA-binding proteins, to target them to specific tRNAs.

## 6. Conclusions

The structural and chemical features of RNA provide excellent solutions for length measurement in biology. In the examples of the RNase P and the T-box riboswitch, structured RNAs act both as the ligand to be measured, and as the measuring devices themselves. Diverse strategies—some selective for sequence, others for structure—are employed to stipulate rules of ligand-ruler recognition, in ways that befit the functions of the RNA devices. Nonetheless, some tried-and-true strategies, such as the interdigitated T-loops for the tRNA elbow, are favored and reused extensively. With our expanding appreciation of the structure of the noncoding transcriptome, novel RNA devices including rulers will be increasingly discovered in nature and created in the laboratory.
